# The Relationship of Conduction Disorder and Prognosis in Patients with Acute Coronary Syndrome

**DOI:** 10.1155/2022/9676434

**Published:** 2022-10-22

**Authors:** Wei-Chieh Lee, Yen-Nan Fang, Tien-Yu Chen, Yun-Yu Hsieh, Yi-Hsuan Tsai, Hsiu-Yu Fang, Po-Jui Wu, Huang-Chung Chen, Ping-Yen Liu

**Affiliations:** ^1^Institute of Clinical Medicine, College of Medicine, National Cheng Kung University, Tainan, Taiwan; ^2^Division of Cardiology, Department of Internal Medicine, Chi Mei Medical Center, Tainan, Taiwan; ^3^Division of Cardiology, Department of Internal Medicine, Kaohsiung Chang Gung Memorial Hospital, Chang Gung University College of Medicine, Kaohsiung, Taiwan; ^4^Biostatistics Center, Kaohsiung Chang Gung Memorial Hospital, Kaohsiung, Taiwan; ^5^Division of Cardiology, Department of Internal Medicine, National Cheng Kung University Hospital, College of Medicine, National Cheng Kung University, Tainan, Taiwan

## Abstract

**Objective:**

Conduction disorders with a widened QRS are associated with poor prognosis in patients with acute coronary syndrome (ACS). Conduction disorders include left bundle branch block (LBBB), right bundle branch block (RBBB), and nonspecific intraventricular conduction delay (NICD). Previous studies did not have conflicting results regarding the type of bundle branch block (BBB) with the worst prognosis, and few studies have focused on the prognosis of patients with NICD.

**Methods:**

Patients with ACS were enrolled between January 2005 and December 2019, and their medical history (International Classification of Diseases codes) was obtained from the Chang Gung Research Database. Age, sex, comorbidities, left ventricular ejection fraction (LVEF), and drug use were compared between the patients with and without conduction disorders. The following clinical outcomes were compared between patients with and without conduction disorders: heart failure (HF) hospitalization, cardiovascular (CV) mortality, and all-cause mortality. After propensity score matching, the Kaplan–Meier curve analysis for HF hospitalization, CV mortality, and all-cause mortality were compared among patients with LBBB, RBBB, and NICD.

**Results:**

This study enrolled a total of 33970 participants and involved 3392 and 30578 patients with and without conduction disorders, respectively. Older age and a higher prevalence of comorbidities were noted in patients with conduction disorders. Lower mean LVEF was exhibited in the patients with conduction disorders (with vs. without; 44.64 ± 20.73% vs. 49.85 ± 20.63%; *p* < 0.001). During the 3-year follow-up period, higher incidences of HF hospitalization (21.55% vs. 17.51%; *p* < 0.001), CV mortality (17.98% vs. 12.14%; *p* < 0.001), and all-cause mortality (38.86% vs. 31.15%; *p* < 0.001) were noted in the patients with conduction disorder. After ACS events, 10.0% of patients presented with conduction disorders, with LBBB in 3.3%, RBBB in 6.0%, and NICD in 0.7%. The lowest mean of LVEF was presented in the patients with NICD (LBBB vs. RBBB vs. NICD; 41.00 ± 19.47% vs. 47.73 ± 20.82% vs. 34.57 ± 20.02%; *p* < 0.001). Among the three groups, the highest incidence of HF hospitalization was noted in patients with LBBB after propensity score matching. The lowest incidence of CV and all-cause mortality was observed in patients with RBBB. After adjustment of age, gender, comorbidities, medication, and mean LVEF, those with LBBB had the highest hazard ratio for major adverse cardiovascular events (MACEs) of 1.113 (*p*=0.029; 95% CI = 1.013–1.266).

**Conclusions:**

In the ACS population, patients with conduction delay had a poor prognosis due to a higher prevalence of comorbidities and lower mean LVEF. Among the patients with LBBB, RBBB, and NICD, those with LBBB and NICD had a higher incidence of HF hospitalization, CV mortality, and all-cause mortality. Patients with NICD had the lowest mean LVEF compared to those with LBBB and RBBB. Patients with LBBB had a significantly highest HR of MACE.

## 1. Background

Acute coronary syndrome (ACS) presents with ischemic syndromes, including unstable angina, non-ST-segment elevation myocardial infarction (NSTEMI), and ST-segment elevation myocardial infarction (STEMI), according to changes in electrocardiography (ECG) and/or elevated cardiac biomarkers [1]. It is associated with significant complications, such as heart failure (HF), mitral valve regurgitation, ventricular septal defect, interventricular conduction disorders, and arrhythmia [1]. Conduction disorders are often associated with conduction system dysfunction due to large myocardial necrosis and can be of different types, such as left and right bundle branch blocks (LBBB and RBBB, respectively) and nonspecific intraventricular conduction delay (NICD) [2, 3] Conduction disorders are associated with increased mortality, particularly in patients with MI and HF [3–5].

In NSTEMI, the QRS duration has prognostic implications, and a QRS duration greater than 90 msec is independently associated with an increased risk of cardiovascular disease in the long-term [6]. In patients with MI, new-onset BBB is related to worse short- and long-term prognosis, and patients with LBBB have a higher mortality due to a higher prevalence of comorbidities [7]. Similar to the guidelines regarding new-onset LBBB, new-onset RBBB should be considered as a standard indicator for reperfusion therapy because RBBB is associated with more severe symptoms and higher incidences of complete occlusion of infarction-related arteries [8, 9]. New-onset RBBB is likely to increase long-term mortality, ventricular arrhythmia, and cardiogenic shock, and reperfusion therapy should be considered when persistent ischemic symptoms occur in patients with RBBB, particularly new-onset RBBB [10]. European Society of Cardiology (ESC) guidelines suggest that patients with LBBB should be managed in a manner similar to STEMI patients, regardless of whether the LBBB is previously known, and reperfusion therapy should be considered when persistent ischemic symptoms occur in patients with RBBB [11]. Few studies have focused on the impact of NICD implantation on the prognosis in the patients with ACS.

Herein, we conducted a large cohort study to explore the impact of conduction disorders on the prognosis of patients with ACS and different types of conduction disorders, including LBBB, RBBB, and NICD.

## 2. Methods

### 2.1. Patient Population

Patients with ACS from January 2005 to December 2019 were recruited, and their medical history, including detailed laboratory values, electrocardiographic reports, and drug use, was obtained from the Chang Gung Research Database (CGRD), which is the largest healthcare system in Taiwan.

The inclusion criteria were as follows: patients with age ≥ 18 years, diagnosis of ACS (International Classification of Diseases, Ninth Revision, Clinical Modification [ICD-9-CM] codes 410.xx, 411.xx, and 412.xx, or Tenth Revision [ICD-10] codes I20, I21, and I22), and patients who had electrocardiographic reports. Patients were divided into two groups (with and without conduction disorder), and patients with conduction disorder were further separated into three groups (LBBB, RBBB, and NICD).

Data on general demographics, comorbidities, left ventricular ejection fraction (LVEF), medication use, HF hospitalization, cardiovascular (CV) mortality, and all-cause mortality of patients were obtained and compared among the three groups.

### 2.2. Ethical Statement

This retrospective study was approved for human research by the Institutional Review Committee of Kaohsiung Chang Gung Memorial Hospital (number: 202101055B0) and conformed to the ethical guidelines of the 1975 Declaration of Helsinki.

### 2.3. Definition

The definitions of LBBB (QRS duration ≥130 msec; QS or rS in lead V1; broad R waves in leads I, aVL, V5, or V6; and absent q waves in leads I, V5, and V6) and RBBB (QRS duration ≥130 msec; rsr', rsR', rSR', or qR in leads V1 or V2; and occasionally, a wide and notched R wave and wide S waves in leads I, V5, and V6) are very precise and seek to define the components of a characteristic activation sequence on the ECG [12]. The definition of NICD is a wide QRS (≥130 msec) but without the typical features of LBBB or RBBB [12].

HF hospitalization was defined as admission to emergency department, hospitalization for HF, and the need for intravenous diuretic agent use. CV mortality was defined as CV-related death. All-cause mortality was defined as death from any cause. Major adverse cardiovascular events included HF hospitalization, CV mortality, and all-cause mortality.

### 2.4. Study Endpoint

The study endpoints were HF hospitalization, CV mortality, and all-cause mortality.

We accepted patients as meeting a study endpoint (HF hospitalization, CV mortality, or all-cause mortality) if they were categorized as such by the ICD discharge code.

### 2.5. Statistical Analyses

Data are presented as the mean ± standard deviation or numbers (percentages). The clinical characteristics of the two groups were compared using the independent sample *t*-test and the chi-square test for categorical variables. The clinical characteristics of the three groups were compared using analysis of variance and Fisher's exact test for categorical variables. Propensity score matching was performed among the LBBB, RBBB, and NICD groups to adjust for differences in the baseline characteristics in the matched analysis. Kaplan–Meier curve analysis was performed using the log-rank test for HF hospitalization, CV mortality, and all-cause mortality in the groups during the 3-year follow-up period. After adjustment of age, gender, and comorbidities, multivariate Cox regression analyses on MACE were performed to determine the HR among the groups. The patient without conduction delay was set as reference for HR. Statistical significance was set at *p* < 0.05. All analyses were performed using SAS version 9.4 (SAS Institute. Inc, Cary, NC, USA).

## 3. Results

### 3.1. The Comparison of Baseline Characteristics between the Patients with or without Conduction Disorders

This study enrolled 33970 participants, and their baseline characteristics and renal outcomes are shown in Table 1. In the patients with conduction disorders, older age (with vs. without; 69 ± 13.6 vs. 66 ± 13.8 years old; *p* < 0.001), higher prevalence of male sex (with vs. without; 71.11% vs. 68.96%; *p*=0.010), and a lower body mass index (BMI) were noted. A higher prevalence of peripheral arterial occlusive disease, chronic obstructive pulmonary disease, end-stage renal disease (ESRD), prior gastrointestinal bleeding, and HF was noted in the patients with conduction disorders. A higher prevalence of smoking and valvular heart disease was noted in the patients without conduction disorders. Higher prevalence of ticagrelor and diuretic agents use were observed in patients with conduction disorders. Lower mean LVEF was exhibited in patients with conduction disorder (with vs. without; 44.64 ± 20.73% vs. 49.85 ± 20.63%; *p* < 0.001).

### 3.2. Kaplan–Meier Curve Analysis for HF Hospitalization, CV Mortality, and All-Cause Mortality in the Patients with or without Conduction Disorder during the 3-Year Follow-Up Period

**Figure 1:**
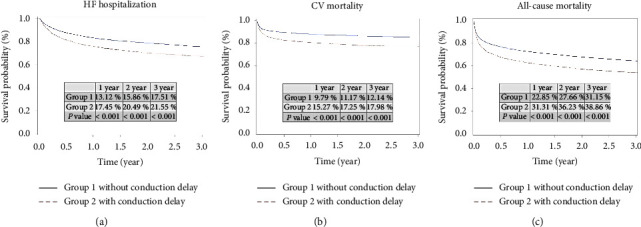
Kaplan–Meier curve analysis for heart failure (HF) hospitalization, cardiovascular (CV) mortality, and all-cause mortality in patients with or without conduction disorders during the 3-year follow-up period. (a): A higher incidence of HF hospitalization is noted in patients with conduction disorders. (with vs. without; 21.55% vs. 17.51%; *p* < 0.001). (b): A higher incidence of CV mortality is noted in patients with conduction disorders. (with vs. without; 17.98% vs. 12.14%; *p* < 0.001). (c): A higher incidence of all-cause mortality is noted in patients with conduction disorders. (with vs. without; 38.86% vs. 31.15%; *p* < 0.001).

During 3-year follow-up period, higher incidences of HF hospitalization (with vs. without; 21.55% vs. 17.51%; *p* < 0.001; Figure 1(a)), CV mortality (with vs. without; 17.98% vs. 12.14%; *p* < 0.001; Figure 1(b)), and all-cause mortality (with vs. without; 38.86% vs. 31.15%; *p* < 0.001; Figure 1(c)) were noted in the patients with conduction disorder.

### 3.3. The Comparison of Baseline Characteristics among the Patients with LBBB, RBBB, and NICD before and after Propensity Score Matching

In the patients with conduction delay (Table 2), LBBB, RBBB, and NICD presented in 3.3%, 6.0%, and 0.7% of patients, respectively. Before propensity score matching, the youngest age, lowest prevalence of male sex, and highest BMI were noted in patients with NICD when compared to patients with LBBB and RBBB. The highest prevalence of diabetes mellitus, hypertension, and ESRD was noted in patients with NICD. The lowest prevalence of HF was noted in patients with RBBB. The lowest use of *β*-blockers and diuretic agents and the highest mean of LVEF were noted in RBBB group (LBBB vs. RBBB vs. NICD; 41.00 ± 19.47% vs. 47.73 ± 20.82% vs. 34.57 ± 20.02%; *p* < 0.001).

After propensity score matching, the mean age, prevalence according to sex, comorbidities, medication use, and mean LVEF did not differ significantly among the three groups.

### 3.4. Kaplan–Meier Curve Analysis for HF Hospitalization, CV Mortality, and All-Cause Mortality among the Patients with LBBB, RBBB, and NICD after Propensity Score Matching during the 3-Year Follow-Up Period

**Figure 2:**
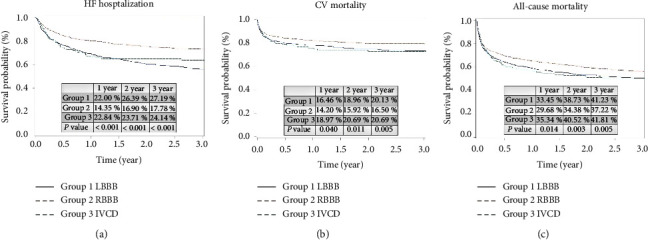
Kaplan–Meier curve analysis for heart failure (HF) hospitalization, cardiovascular (CV) mortality, and all-cause mortality among the patients with left bundle branch block (LBBB), right bundle branch block (RBBB), and nonspecific intraventricular conduction delay (NICD) after propensity score matching during the 3-year follow-up period. (a): Between three groups, highest incidence of HF hospitalization is noted in patients with LBBB after propensity score matching (LBBB vs. RBBB vs. NICD; 27.91% vs. 17.78% vs. 24.14%; *p* < 0.001). (b): Between three groups, lowest incidence of CV mortality is noted in patients with RBBB after propensity score matching (LBBB vs. RBBB vs. NICD; 20.13% vs. 16.50% vs. 20.69%; *p*=0.005). (c): Between three groups, lowest incidence of all-cause mortality is noted in the patients with RBBB after propensity score matching (LBBB vs. RBBB vs. NICD; 41.23% vs. 37.22% vs. 41.81%; *p*=0.005).

Among three groups, highest incidence of HF hospitalization (LBBB vs. RBBB vs. NICD; 27.91% vs. 17.78% vs. 24.14%; *p* < 0.001) was noted in the patients with LBBB after propensity score matching (Figure 2(a)). The lowest incidence of CV mortality (LBBB vs. RBBB vs. NICD; 20.13% vs. 16.50% vs. 20.69%; *p*=0.005; Figure 2(b)) and all-cause mortality (LBBB vs. RBBB vs. NICD; 41.23% vs. 37.22% vs. 41.81%; *p*=0.005; Figure 2(c)) presented in the patients with RBBB.

### 3.5. Multivariate Cox Regression Analyses for MACE

Multivariate Cox regression for MACE among the groups is shown in Table 3. Those without conduction were set as reference. Those with conduction delay had an HR of 1.058 (*p*=0.127; 95% confidence interval [CI] = 0.984-1.137) after adjustment of age, gender, and comorbidities. Those with LBBB had an HR of 1.113 (*p*=0.029; 95% CI = 1.013–1.266). Those with RBBB had an HR of 1.011 (*p*=0.827; 95% CI = 0.919–1.111). Those with RBBB had an HR of 1.062 (*p*=0.611; 95% CI = 0.841–1.342).

## 4. Discussion

Interventricular conduction disorders are among the complications of acute myocardial infarction (MI) and can be of different types, including LBBB, RBBB, and NICD. Previous studies did not have conflicting results regarding the type of BBB with the worst prognosis [7–10]. In this cohort study, 10.0% of patients presented conduction disorder after ACS events, with LBBB, RBBB, and NICD in 3.3%, 6.0%, and 0.7% of patients, respectively. Patients with conduction disorders had a worse prognosis for HF hospitalization, CV mortality, and all-cause mortality. Among patients with conduction disorders, those with RBBB had a lower incidence of HF hospitalization, CV mortality, and all-cause mortality. Among patients with LBBB, RBBB, and NICD, a higher mean LVEF was observed in patients with RBBB. The prognosis of patients with NICD also weakened the prognosis of patients with ACS, similar to the prognosis of patients with ACS and LBBB, when compared to patients with RBBB or without conduction disorders. The patients with LBBB had a significantly higher HR of MACE than that of the patients without conduction disorders.

Two meta-analyses confirmed that patients with ACS and RBBB had the highest mortality (in-hospital and long-term), but there was considerable heterogeneity across the included studies [10, 11]. RBBB runs in the interventricular septum, which is supplied by the first septal branch separated from the left anterior descending artery, and new-onset RBBB may be caused by the complete occlusion of the infarct-related artery [13]. A previous study indicated that new-onset RBBB was a significant independent risk factor for predicting adverse in-hospital events [8, 9, 14]. In our study, patients with RBBB had a lower incidence of HF hospitalization, CV mortality, and all-cause mortality than patients with LBBB and NICD. Our results were different as our study enrolled the patients with ACS and not only MI. However, the presence of RBBB may confound the diagnosis of STEMI and delay reperfusion therapy in patients with MI, which can also influence the clinical outcomes. In our study, patients with RBBB still had a poorer prognosis than those without conduction disorders.

LBBB masks ST-segment shifts, repolarization phase changes, or Q waves and can present with acute MI with either STEMI equivalent or NSTEMI equivalent physiology [15]. Therefore, the presence of new or presumably new LBBB in a patient with symptoms compatible with AMI was considered a class I indication for emergent reperfusion therapy for STEMI equivalent [1, 16]. LBBB occurs in up to 30% of patients with HF and is associated with poor prognosis due to cardiac comorbidities and myocardial dysfunction [17]. In patients with ACS and LBBB, it is reasonable that such a population has a higher incidence of HF hospitalization and mortality.

A different definition of NICD is the existence of a widened QRS without the features of RBBB or LBBB and a QRS duration of ≥110 msec in adults [18]. Only a few studies have focused on NICD, and its pathophysiology is complex and reflects intramyocardial conduction delay due to cardiomyopathy [19]. NICD is also related to increased long-term mortality and the future occurrence of atrial fibrillation and HF [19–21]. In patients with conduction disorders, patients with NICD had a similar incidence of HF hospitalization, CV mortality, and all-cause mortality when compared to patients with LBBB. In our study, patients with NICD had the worst mean LVEF compared to those with other conduction disorders.

### 4.1. Study Limitations

This study had several limitations. First, the study design was retrospective, and all information was obtained from medical records. Second, the ECG findings were obtained from the report by one cardiologist, and the QRS length was not available. Nevertheless, this study provides valuable information regarding the relationship between conduction disorders and clinical outcomes in patients with ACS. Third, in our health care system, the patients without clinical events need to be transferred to local healthcare system from medical center. Therefore, the follow-up period was limited within three years.

## 5. Conclusions

In the ACS population, patients with conduction delay had a poor prognosis due to a higher prevalence of comorbidities and lower mean LVEF. Among the patients with LBBB, RBBB, and NICD, those with LBBB and NICD had a higher incidence of HF hospitalization, CV mortality, and all-cause mortality. Patients with NICD had the lowest mean LVEF compared to those with LBBB and RBBB. Patients with LBBB had a significantly highest HR of MACE. Therefore, we need to pay more attention for HF treatment in the patients with conduction disorders, especially LBBB and NICD.

## Figures and Tables

**Table 1:** Baseline characteristics and clinical outcomes in the patients with and without conduction disorder.

	With conduction disorder	Without conduction disorder	*p*-Value
Number general demographics	3392	30578	
Age (years)	69 (13.6)	66 (13.8)	<0.001
Male sex (%)	2412 (71.11)	21088 (68.96)	0.010
BMI (kg/m^2^)	24.84 (4.38)	25.04 (4.39)	0.024

Comorbidities			
Diabetes mellitus (%)	1279 (37.71)	11079 (36.23)	0.090
Hypertension (%)	1939 (57.16)	17089 (55.89)	0.155
PAOD (%)	47 (1.39)	213 (0.70)	<0.001
COPD (%)	214 (6.31)	1511 (4.94)	<0.001
ESRD (%)	480 (14.15)	2425 (7.93)	<0.001
Smoking (%)	387 (11.41)	4590 (15.01)	<0.001
Liver cirrhosis (%)	44 (1.30)	486 (4.59)	0.193
Prior GI bleeding (%)	407 (12.00)	3180 (10.40)	0.004
Prior stroke (%)	227 (6.69)	2085 (6.82)	0.782
HF (%)	1118 (32.96)	7097 (23.21)	<0.001
Valvular heart disease (%)	159 (5.99)	1815 (8.13)	<0.001

Medication			
Antiplatelet agent			
Aspirin (%)	2032 (59.91)	19709 (64.45)	<0.001
Clopidogrel (%)	1596 (47.05)	15684 (51.29)	<0.001
Ticagrelor (%)	556 (16.39)	4381 (14.33)	0.001
Prasugrel (%)	16 (0.47)	145 (0.47)	0.984
ACEI/ARB/Entresto (%)	1727 (50.91)	15972 (52.23)	0.144
*β*-blocker (%)	1890 (55.72)	17340 (56.71)	0.271
Diuretic (%)	864 (25.47)	5948 (19.45)	<0.001
Lipid-lowering agents (%)	1784 (52.59)	16329 (53.40)	0.372

Left ventricular performance			
Mean LVEF (%)	44.64 (20.73)	49.85 (20.63)	<0.001

F/U period (years)	1.8 (1.5)	2.9 (2.6)	<0.001

Data are expressed as mean (standard deviation) or as number (percentage). Abbreviation: BMI: body mass index; PAOD: peripheral arterial occlusive disease; COPD: chronic obstructive pulmonary disease; ESRD: end-stage renal disease; GI: gastrointestinal; HF: heart failure; ACEI: angiotensin-converting enzyme inhibitor; ARB: angiotensin receptor blocker; MRA: mineralocorticoid receptor antagonist; LVEF: left ventricular ejection fraction; F/U: follow-up.

**Table 2:** Baseline characteristics and clinical outcomes in the patients with LBBB or RBBB or NICD.

	Before propensity score matching				After propensity score matching			
	LBBB	RBBB	NICD	*p* value	LBBB	RBBB	NICD	*p*-Value
Number general demographics	1118	2042	232		125	125	125	
Age (years)	70 (13.1)^a^	69 (13.9)^b^	66 (13.6)^c^	<0.001	66 (14.1)	66 (14.4)	66 (14.1)	0.975
Male sex (%)	364 (32.56)^a^	570 (27.91)^b^	46 (19.83)^c^	<0.001	104 (83.20)	102 (81.6)	102 (81.6)	0.930
BMI (kg/m^2^)	24.46 (4.34)^a^	24.95 (4.40)^b^	25.74 (4.25)^c^	<0.001	25.81 (4.91)	25.90 (4.24)	25.70 (4.34)	0.687

Comorbidities								
Diabetes mellitus (%)	434 (38.82)^a^	733 (35.90)^a^	112 (48.28)^b^	<0.001	63 (50.40)	64 (51.20)	66 (52.80)	0.928
Hypertension (%)	629 (56.26)^a^	1156 (56.61)^a^	154 (66.38)^b^	0.013	83 (66.40)	86 (68.80)	87 (69.60)	0.852
COPD (%)	61 (5.46)	214 (6.31)	13 (5.60)	0.272	5 (4.00)	5 (4.00)	6 (4.80)	0.937
ESRD (%)	184 (16.46)^a^	246 (12.05)^b^	50 (21.55)^a^	<0.001	32 (25.60)	27 (21.60)	29 (23.20)	0.754
Smoking (%)	118 (10.55)	236 (11.56)	33 (14.22)	0.263	13 (10.40)	12 (9.60)	15 (12.00)	0.822
Prior GI bleeding (%)	148 (13.24)	228 (11.17)	31 (13.36)	0.185	16 (12.80)	9 (7.20)	15 (12.00)	0.300
Prior stroke (%)	89 (7.96)	122 (5.97)	16 (6.90)	0.101	7 (5.60)	7 (5.60)	5 (4.00)	0.801
HF (%)	483 (43.20)^a^	533 (26.10)^b^	102 (43.97)^a^	<0.001	55 (44.00)	65 (52.00)	58 (46.40)	0.430
Valvular heart disease (%)	64 (6.96)	84 (5.43)	11 (5.95)	0.209	—	—	—	—

Medication								
Antiplatelet agent								
Aspirin (%)	689 (61.63)	1200 (58.77)	143 (61.64)	0.250	82 (65.60)	91 (72.80)	82 (65.60)	0.371
Clopidogrel (%)	547 (48.93)	937 (45.89)	112 (48.28)	0.243	64 (51.20)	72 (57.60)	66 (52.80)	0.572
Ticagrelor (%)	190 (16.99)	336 (16.45)	30 (12.93)	0.312	21 (16.80)	20 (16.00)	15 (12.00)	0.522
ACEI/ARB/Entresto (%)	600 (53.67)	1009 (49.41)	118 (50.86)	0.073	67 (53.60)	72 (57.60)	67 (53.60)	0.764
*β*-blocker (%)	662 (59.21)^a^	1096 (53.67)^b^	132 (56.90)^a,b^	0.010	81 (64.80)	84 (67.20)	81 (64.80)	0.899
MRA or diuretic (%)	358 (32.02)^a^	433 (21.20)^b^	73 (31.47)^a^	<0.001	44 (35.20)	46 (36.80)	46 (36.80)	0.955
Lipid-lowering agents (%)	594 (53.13)	1058 (51.81)	132 (56.90)	0.308	74 (59.20)	70 (56.00)	80 (64.00)	0.431

Left ventricular performance								
Mean LVEF (%)	41.00 (19.47)^a^	47.73 (20.82)^b^	34.57 (20.02)^c^	<0.001	35.39 (16.06)	35.59 (18.11)	35.05 (19.99)	0.549

F/U period (years)	1.4 (1.1)^a^	2.0 (1.7)^b^	1.6 (1.4)^c^	<0.001	—	—	—	—

Data are expressed as mean (standard deviation) or as number (percentage). Different letters (a, b) associated with different groups indicate significant difference (at 0.05 level) by Bonferroni multiple comparison procedure. Abbreviation: LBBB: left bundle branch block; RBBB: right bundle branch block; NICD: nonspecific intraventricular conduction delay; BMI: body mass index; PAOD: peripheral arterial occlusive disease; COPD: chronic obstructive pulmonary disease; ESRD: end-stage renal disease; GI: gastrointestinal; HF: heart failure; ACEI: angiotensin-converting enzyme inhibitor; ARB: angiotensin receptor blocker; MRA: mineralocorticoid receptor antagonist; LVEF: left ventricular ejection fraction; F/U: follow-up.

**Table 3:** Multivariate Cox regression analyses of predictors of MACE.

Variables	HR	95% CI	*p*-Value
Without conduction delay	Reference		
With conduction delay	1.058	0.984–1.137	0.127
LBBB	1.133	1.013–1.266	0.029
RBBB	1.011	0.919–1.111	0.827
NICD	1.062	0.841–1.342	0.611

Abbreviation: MACE: major adverse cardiovascular event; HR: hazard ratio; CI: confidence interval; LBBB: left bundle branch block; RBBB: right bundle branch block; NICD: nonspecific intraventricular conduction delay.

## Data Availability

The study data are available from the corresponding author upon reasonable request.
